# Genetic Diversity of the ORF5 Gene of Porcine Reproductive and Respiratory Syndrome Virus Isolates in Southwest China from 2007 to 2009

**DOI:** 10.1371/journal.pone.0033756

**Published:** 2012-03-20

**Authors:** Gefen Yin, Libo Gao, Xianghua Shu, Guishu Yang, Shuhao Guo, Wengui Li

**Affiliations:** College of Animal Science and Technology, Yunnan Agricultural University, Kunming, China; Texas Veterinary Medical DIagnostic Laboratory-Amarillo, Texas A&M System, United States of America

## Abstract

To gain insight into the molecular epidemiology and possible mechanisms of genetic variation of porcine reproductive and respiratory syndrome (PRRS) in Yunnan Province of China, the ORF5 gene of 32 PRRSV isolates from clinical samples collected from 2007 to 2009 were sequenced and analyzed. Nucleotide and amino acid analyses were carried out on 32 isolates and representative strains of the North American genotype, European genotype and two representative Chinese isolates. Results revealed that these isolates share 86.9–99.0% nucleotide and 87.5–98.0% amino acid identity with VR-2332 the prototypical North American PRRSV, 61.7–62.9% and 54.3–57.8% with Lelystad virus (LV) the representative strain of European genotype, 91.2–95.4% and 90.0–94.5% with CH-1a that was isolated in mainland China in 1996, 88.1–99.3% and 85.5–99.0% with JX-A1 the representative strain of High pathogenic PRRSV in China, and 86.2–99.8% and 85.5–100.0% between isolated strains of different years, respectively. Phylogenetic analysis revealed that all 32 PRRSV isolates belonged to the North American genotype and were further divided into two different subgenotypes. Subgenotype 1 comprised twenty two Yunnan isolates which divided into two branches. Subgenotype 2 comprised ten isolates which closely related to the RespPRRS vaccine and its parent strain VR-2332. The functional domains of GP5 such as the signal peptide, ectodomain, transmembrane regions and endodomain were identified and some motifs in GP5 with known functions, such as primary neutralizing epitope (PNE) and decoy epitope were also further analyzed. Our study shown the great genetic diversity of PRRSV in southwest China, rendering the guide for control and prevention of this disease.

## Introduction

Porcine reproductive and respiratory syndrome (PRRS) is the most economically significant disease of swine worldwide. The disease mainly causes premature delivery, miscarriage, stillbirth, mummified fetuses, severe pneumonia, edema and conjunctivitis in pigs. Typical PRRS is also called “blue ear” disease due to a representative symptom of the infected piglets [1]. In addition, coinfection and secondary infection causes significantly higher mortality rate [2].

PRRS first emerged in late 1987 in the United States and three years later in Europe. Two genotypes are recognized for Porcine reproductive and respiratory syndrome virus (PRRSV), the North American genotype and the European genotype, as represented by the prototypes VR-2332 and Lelystad virus (LV), respectively [3]. In mainland China, the North American genotype was first reported in 1996 and it has spread throughout the country with considerable genetic variation [4].

PRRSV belongs to the family Arteriviridae in the order Nidovirales, a family of positive-sense, single stranded linear RNA viruses. PRRSV is an enveloped arterivirus, 50–60 nm in size. The genome is about 15 kb in length which contains nine ORFs. The 3′ end of the genome encodes four membrane-associated glycoproteins (GP2, GP3, GP4 and GP5, encoded by subgenomic mRNAs 2a, 3, 4 and 5), two unglycosylated membrane proteins (E and M, encoded by subgenomic mRNAs 2b and 6) and a nucleocapsid protein [5], [6]. The 5′ untranslated region (5′ UTR) includes stem loop 2 which is a key structural element for PRRSV replication and subgenomic mRNA synthesis [7]. A recent study revealed that a 51 amino acid polypeptide referred to as ORF5a was encoded by an alternative ORF of the subgenomic mRNA encoding GP5, and a similar ORF was present as an alternative reading frame in all PRRSV subgenomic RNA5 genes and in all other arteriviruses [8].

The GP5 protein is the primary envelope protein, a glycoprotein of approximately 200 amino acids with an apparent molecular mass of 26 kDa. There are 2–4 glycosylation sites, a 31 amino acid signal peptide and 6 antigenic determinants which induce neutralizing antibodies included in GP5. The presence of a major neutralization epitope in the N-terminal ectodomain implied that GP5 is involved in receptor recognition [9].

As the main candidate protein for development of subunits vaccine, also due to its immunological significance and polymorphic nature, GP5 has been the target for analysis of genetic diversity of PRRSV [10]. In the present study, ORF5 gene of 32 PRRSV isolates from clinical samples in Yunnan (southwest China) during the period of 2007–2009 were sequenced and analyzed to better understand the molecular epidemiology of PRRS.

## Materials and Methods

### 1. Sample collection

A total of 810 clinical samples including lung, blood and semen were sampled from different swine herds that experienced outbreaks of severe reproductive failure in pregnant sows and respiratory problems in sucking and post-weaning piglets in Yunnan Province, southwestern China from 2007 to 2009.

Sampling procedures were approved by the Animal Care and Use Committee of Yunnan Province, China, which does not issue a number to any animal study. Our sampling processes were assisted by local authorities and veterinarians. Blood was sampled in each PRRS suspect cases, boar semen was collected with an artificial vagina and lung samples were collected during necropsy. Pieces of the tissues from pigs were homogenized for RNA extraction and virus isolation, remaining samples were kept at −70°C.

### 2. RNA Extraction and Reverse Transcription

Total RNA was extracted from the tissue homogenates using Qiagen RNeasy Mini kit. The extracted RNA was dissolved in RNase-free pure water and used immediately for reverse transcription in a final 20 µl reaction volume. Reaction mixtures consisted of 6 µl RNA solution, 0.5 µl 10 mM Y4 reverse primer, 0.5 µl (40 U) RNAase inhibitor (Toyobo Co. Japan), 4 µl 5×MMLV Buffer and 1 µl 10 mM dNTPs. Before 1 µl (100 U) MMLV (Takara Co. Dalian, China) was added, the mix was incubated at 70°C for 5 min and then placed on ice for 5 min. Reverse transcription was conducted at 42°C for 1 hour, with a final termination at 70°C for 15 min.

### 3. Primer design and RT-PCR

Based upon multiple sequence alignment, a set of PRRSV primers Y3 and Y4 were designed for RT-PCR assay. The sense primer Y3 was 5′-cca tgt tgg aga aat gct tg-3′ and the reverse primer Y4 was 5′-cgg ccg cga ctc acc ttt ag-3′. The cycling conditions were 95°C for 5 min, followed by 30 cycles of 94°C for 40 sec, 56°C for 40 sec, 72°C for 60 sec, and a final extension at 72° C for 10 min. This yielded a 708 bp PCR product containing complete ORF5 gene of PRRSV.

### 4. Cloning, sequencing and phylogenetic analysis of PCR products

The PCR products were purified using PCR purification kit (TaKaRa Co. Dalian, China) and cloned into the pMD18-T vector (TaKaRa Co. Dalian, China). The plasmid was then used to transform E. Coli DH5α. The plasmids were extracted and the inserts sequenced at Sangon Biological Engineering Company (Shanghai, China). The 708 nt consensus sequences were aligned using DNAman (version 6.0, Lynnon Co.). Genetic distances between PRRSV sequences were calculated with the DNAStar software (Hitachi, version 7.1, Madison WI, USA). Phylogenetic and molecular evolutionary genetic analyses were conducted using the neighbor-joining method with MEGA (Version 4,Tamura, Dudley, Nei, and Kumar 2007). A total of 29 North American genotypes and 1 European genotype were used as references in the analysis (Table 1).

**Table 1:** Representative PRRSV Strains used in the phylogenetic and sequence analyses.

NO.	Name	Area	Year	Accession No.
**1**	LV	Netherlands	1993	M96262
**2**	VR-2332	USA	1995	U87392
**3**	Clone20	China:Hubei	2003	FJ899592
**4**	4139	USA:MN	2005	EU756000
**5**	RespPRRS	USA	2005	AF066183
**6**	SCQ	China:Sichuan	2006	DQ379479
**7**	BHA	China:Guangxi	2006	EF104600
**8**	CC-1	China:Jilin	2006	EF153486
**9**	HB-3(cz)	China:Hebei	2006	EU478435
**10**	5554	Canada:MB	2006	EU756566
**11**	JSyx	China:Jiangsu	2006	EU939312
**12**	JX-A1*	China:Jiangxi	2006	EF112445
**13**	GZZB	China:Guizhou	2007	EU140611
**14**	JX3	China:Jiangxi	2006	EU213137
**15**	Hainan-2	China:Hainan	2007	EF398052
**16**	GDB11	China:Guangdong	2007	GU980181
**17**	pJX148	China:Jiangxi	2007	EF488048
**18**	HBAN1	China:Hubei	2008	EU371943
**19**	4034-2-V-2008	South Korea	2008	FJ972733
**20**	JN-HS	China: Shandong	2008	HM016158
**21**	FJ07B	China:Fujian	2008	GU358694
**22**	CQJ-1	China: Chongqing	2008	FJ919335
**23**	AHXS4	China:Hubei	2008	EU399857
**24**	JX5	China:Jiangxi	2006	EU213139
**25**	GZJL	China: Guizhou	2009	FJ947000
**26**	BZHM	China:Shandong	2009	GU977226
**27**	BB0907	China:Guangxi	2009	HQ315835
**28**	5112HCM	Viet Nam:Ho Chi Minh	2010	HQ700878
**29**	Ch-1a#	China:Beijing	1996	AY032626
**30**	WUH1	HuBei	2007	EU187484

* JX-A1 is the representative strain of HP-PRRSV in China since 2006;

# Ch-1a is the first PRRSV isolate in mainland China in 1996.

### 5. Amino acid analysis of GP5

To get a better understanding of the genetic relationship and evolution of PRRSV in Yunnan from 2007 to 2009, phylogenetic analysis was carried out based on the sequences of the ORF5 gene of Yunnan Province isolated PRRSV strains, together with some North American genotype isolates from China and other countries. In particular, some known functional domains of GP5 such as the signal peptide, ectodomain, transmembrane regions and endodomain were identified, some motifs in GP5 like primary neutralizing epitope (PNE) and decoy epitope were also analyzed according to a previous report [11].

## Results

### 1. RT-PCR survey of clinical samples

PRRSV was detected in 51 (10 strains in 2007, 25 strains in 2008 and 16 strains in 2009) out of 810 clinical samples by RT-PCR, the total positive rate was 6.3% (51/810).

### 2. Sequence and phylogenetic analyses of ORF5

51 strains sequences were designated in order of isolation from YN07-00 to YN09-50. A further blast-search using the public database in GenBank of the amplification fragment found 19 sequences (No. 7, 9, 15, 16, 21, 22, 25, 26,27,30,32, 33, 34, 36, 41, 45, 46, 48, 49)shared identical sequences and were removed, so that finally 32 Yunnan strains were identified in this study. These sequences for GP5 genes of individual isolates were deposited in GenBank (Table 2).

**Table 2:** Information of 32 PRRSV isolated in Yunnan Province, China.

No.	Isolate	Isolation year	Genebank accession No.	No.	Isolate	Isolation year	Genebank accession No.
**1**	yn07-0	2007	JF920761	**17**	yn08-20	2008	JF920771
**2**	YN-1	2007	FJ151490	**18**	yn08-23	2008	JF920774
**3**	YN-2	2007	FJ361883	**19**	yn08-24	2008	JF920775
**4**	YN-3	2007	FJ361884	**20**	yn08-28	2008	JF920779
**5**	YN-4	2007	FJ361885	**21**	yn08-29	2008	JF920780
**6**	YN-5	2007	FJ361886	**22**	yn08-31	2008	JF920782
**7**	YN-6	2007	FJ361887	**23**	yn09-35	2009	JF920786
**8**	YN-8	2007	FJ361889	**24**	yn09-37	2009	JF920788
**9**	YN-10	2008	FJ361891	**25**	yn09-38	2009	JF920789
**10**	yn08-11	2008	JF920762	**26**	yn09-39	2009	JF920790
**11**	yn08-12	2008	JF920763	**27**	yn09-40	2009	JF920791
**12**	yn08-13	2008	JF920764	**28**	yn09-42	2009	JF920793
**13**	yn08-14	2008	JF920765	**29**	yn09-43	2009	JF920794
**14**	yn08-17	2008	JF920768	**30**	yn09-44	2009	JF920795
**15**	yn08-18	2008	JF920769	**31**	yn09-47	2009	JF920798
**16**	yn08-19	2008	JF920770	**32**	yn09-50	2009	JF920801

**Figure 1:**
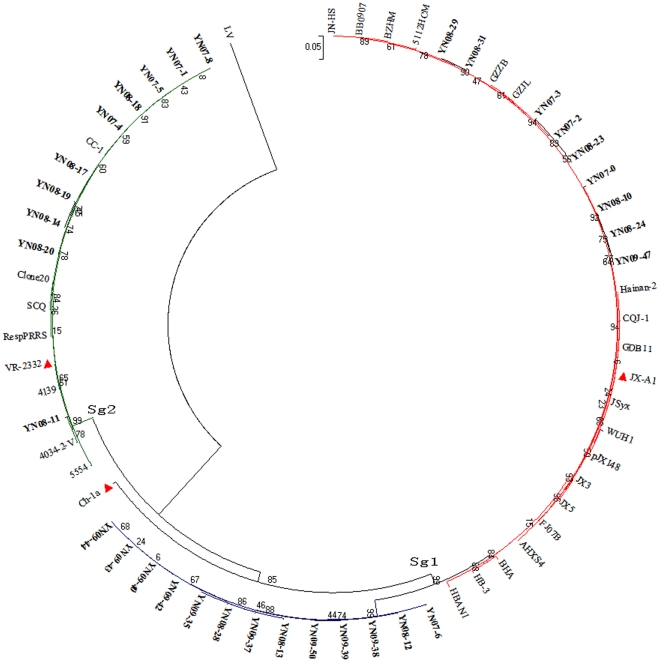
Phylogenetic relationship of PRRSVs. A neighbor-joining tree was constructed based on sequences of the ORF5 gene of PRRSV, with bootstrap values calculated from 1,000 replicates. Subgroups are marked with different colors and the strains isolated in this study are indicated in bold.

Further sequence analysis identified all 32 isolated strains belonging to North American genotype. Compared with representative strain VR-2332 for the North American genotype, LV for the European genotype, CH-1a and JX-A1 for Chinese isolates, nucleotide homologies ranged from 86.9–99.0%, 61.7–62.9%, 91.2–95.4% and 88.1–99.3%, respectively, and 86.2–99.8% between different years isolated strains (Table 3). A phylogenetic tree was constructed using the neighbor-joining method based on sequences of 32 isolates of PRRSV from Yunnan Province and 29 references PRRSV sequences from China and other countries. As shown in Figure 1, all 32 PRRSV isolates were divided into two different subgenotypes. Subgenotype 1 comprised twenty two Yunnan isolates which divided into two branches. One branch include 13 strains which all the strains isolated from Yunnan and the other include nine Yunnan isolate, along with JX-A1 the representative strain of HP-PRRSV in China since 2006 and other related PRRSV. Subgenotype 2 comprised ten isolates that were highly homologous to each other and closely related to the RespPRRS vaccine and its parent strain VR-2332.

**Table 3:** Analysis of nucleotide and amino acid identity of ORF5 among 32 PRRSV isolates and with represent PRRSV isolates LV, VR-2332, RespPRRSV, Ch-1a, and highly pathogenic strain JX-A1.

Isolation Years(No.)		2007	2008	2009	LV	VR-2332	RespPRRS	JX-A1	Ch-1a
2007(9)	nt	86.9–99.8	86.2∼99.8	86.2∼99.8	61.7∼62.9	87.6∼99.0	87.2∼99.3	88.4∼98.8	91.5∼94.9
	aa	86.5–100.0	85.5∼99.5	86.0∼99.5	55.3∼57.3	88.0∼98.0	87.0∼99.0	86.0∼97.5	90.5∼92.5
2008(13)	nt	-	86.2∼99.8	86.2∼99.8	61.7∼62.9	86.9∼99.0	87.2∼99.3	88.1∼99.3	91.2∼95.4
	aa	-	85.5∼99.5	86.0∼100.0	54.3∼57.8	87.5–98.0	86.5∼99.0	85.5∼99.0	90.0∼93.5
2009(10)	nt	-	-	94.5–99.8	61.9–62.2	86.9∼89.4	87.1∼89.1	93.7∼99.2	92.9∼95.2
	aa	-	-	94.0∼99.5	56.3∼57.3	87.5∼89.0	86.5∼88.0	93.0∼99.0	92.5∼94.5

### 3. Amino acid analysis of the GP5

**Figure 2:**
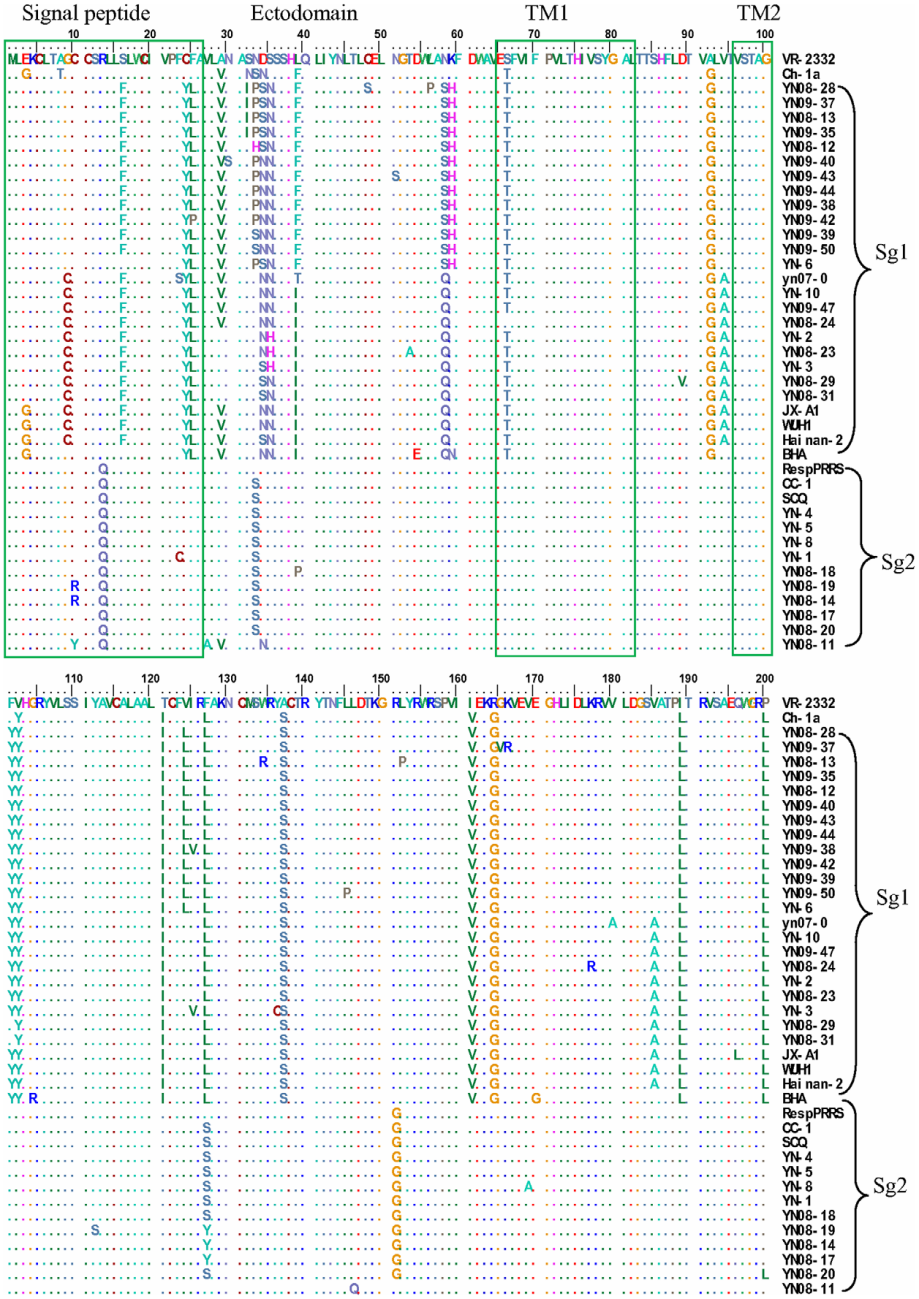
Analysis and comparison of amino acid mutations in ORF5 gene of 32 Chinese isolates and 9 reference strains. Dots (.) indicate the same amino acids as in VR2332 and substitutions are indicated by the amino acid letter codes. The functional domains indicated by color boxes are reported according to Zhou et al. [11].

To investigate the amino acid difference among the two subgenotype isolates, the GP5 amino acid sequences of 32 PRRSV isolates were aligned, together with some North American genotype isolates from China and other countries. Multiple alignments of GP5 sequences of Chinese PRRSV isolates indicated that all 32 isolates encode a GP5 protein of 200 amino acid residues, but that substitutions were extensive (Fig. 2).

Pairwise comparison revealed 85.5–100.0% deduced amino acid similarity between the 32 Yunnan PRRSV isolates and shared 87.5–98.0% amino acid identity with the prototypical North American PRRSV VR-2332, 54.3–57.8% with strain LV the European type, 85.5–99.0% with JX-A1, and 90.0–94.5% with CH-1a (Table 3).

Compared with those in subgenotypes 2, Yunnan PRRSV strains in subgenotypes 1 were found to be highly variable in the primary neutralizing epitope (PNE) and contained an amino acid mutation (F39/L39→I39). To differentiate these isolates of different subgenotypes, the unique or concurrent amino acid mutations were observed. Three (L39→F39/I39, N58→S58/Q58, K59→H59; and G9→C9, V94→A94, V185→A185) and two (R13→Q13, R151→G151) unique mutations observed in subgenotype 1 and subgenotype 2, respectively. Other representative concurrent amino acid mutations (S16→F16, C24→Y24, A29→V29, S66→T66, A92→G92, 101F→101Y, 102V→102Y, T121→I121, A137→S137, I161→V161, R164→G164, I189→L189, P200→L200) were also found in two subgenotypes.

## Discussion

PRRS has become widespread since its initial outbreak in mainland China at the end of 1995, the infection rate of Chinese swine herds has risen to 90% [12]. An increasing number of field PRRSV strains have been isolated from different regions of China. However, all described PRRS viruses in China, including the HP-PRRSV isolates almost simultaneously emerged both in China and Vietnam in 2006, are of the North American genotype [13]. Recently, there was report that European genotype PRRSV isolates were detected in swine herds both with and without clinical symptoms in China. Further sequence analysis revealed that they shared 87.0–91.5% and 58.0–58.2% identity with the European and North American genotype prototypic strains LV and VR-2332, respectively [12]. The coexistence of both genotypes PRRSV in Chinese swine herds provides a suitable environment for developing new viral biological characteristics. Meanwhile, it complicates the prevention and control of PRRSV in China.

ORF5 gene has been considered to be one of the least conserved ORFs of PRRSV with respective sequence variability of up to 17%, which lead to a high degree of genetic polymorphism under constant selective pressure even within the same genotype, and the emergence of acute PRRS or highly variable PRRSV. The amino acid substitutions of the GP5 protein between the PRRSV isolates and representatives are mostly observed in the heterogenic signal sequence and the two hyper variable regions [14], [15]. Studies based on ORF5 gene sequences of Chinese isolates have revealed extensive genetic diversity, even in highly pathogenic PRRSV that emerged in 2006 [11], [16].

GP5 of PRRSV is associated with virus neutralization. Three B cell epitopes, two T cell epitopes exists in the North American genotype, and at least two of these epitopes are neutralization epitopes. The PNE of both North American and European PRRSV strains are located in the middle of the GP5 ectodomain, which previously identified as S37H(F/L)QLIYN or S37H(F/L)QLIYN LTLCELNG. The PNE plays an important role in viral neutralization, and the residues H38L39/F39 in this domain are considered to be the critical amino acids of the neutralizing epitope [17], [18]. In this study, analysis of PNE found that the key antibody combined site (L/F)_39_ were mutation to I39 (isolates YN-2, YN-3, YN08-23, YN08-24, YN08-29, YN08-31, YN09-47), T39 (YN07-0) or P39 (YN-1), also amino acid mutations C48→S48 (YN08-28) and N51→S51 (YN09-43) observed in Yunnan isolates. These mutations likely lead to the failure of immunization in Yunnan Province. Nevertheless, further studies are necessary to provide additional evidence. In consideration of that the immunogenicity and specificity of the linear neutralization epitopes are not only determined by the amino acid sequence, but also by conformational effects that are probably induced by amino acid changes upstream or downstream of the epitope [19].

The epitope comprised of A27/V27LVN near the PNE is the main antibody recognition site for epitope A. This epitope may also act as a decoy, which elicit most of the antibodies against GP5 and delay the introduction of neutralizing antibodies for at the least 3 weeks [18]. Among GP5 gene of 32 Yunnan isolates, this decoy epitope was conserved in Subgenotype group 1, whereas nine of ten strains in Subgenotype group 2 contained one amino acid mutation at position 29 (V→A). The replacement of these four amino acids considerably reduced the recognition of this epitope.

The widely use of modified live-attenuated vaccines have reduced the incidence and severity of PRRS outbreaks in many countries. Nevertheless, live PRRSV vaccine virus exhibits an untoward tendency to spread and reversion towards virulence . The periodic identification of vaccine-like isolates poised the challenge for detection and characterization of vaccine-like isolates and field isolates of PRRSV [20]–[22]. Comparisons between the vaccine virus and its parental virus showed that ORF5 codon position 13, 151 and ORF6 codon position 15 play a key role in denaturing VR2332 to RespPRRSV [23], [24]. When VR-2332 was attenuated to the RespPRRS/Repro vaccine strain, R13 and R151 were altered to Q13 and G151, respectively [25]. Since R13Q was included in the signal peptide, this mutation may also have an effect on transfer of GP5. It has been reported that R151G mutation has an effect on hydrophobic areas and reduces the virulence of Czech strain V502 after 152 passages that resulted in this mutation [26]. In this study, R13Q and R151G were observed in nine of ten isolates in subgenotype 2, including a middle virulent strain YN-1 that has been isolated and identified, nevertheless there is no evidence to prove that the field isolates are responsible for the reversion of the modified live PRRS vaccine.

In summary, nucleotide and amino acid sequence analysis of GP5 of 32 PRRSV strains isolated in Yunnan from 2007 to 2009 revealed the prevalence of two subgenotypes of PRRSV in this region. All strains isolated had divergence obviously from the first Chinese isolate Ch-1a, and ten isolates (subgenotype 2) were highly related to the vaccine strain. The occurrence of these vaccine strain related isolates is presumably related to the extensive use of the attenuated modified live PRRS vaccine since live PRRSV vaccines can revert to virulence. In addition, our study determined that a branch of subgenotype 1 is entirely made up of Yunnan isolates, which raising the possibility that PRRSV prevalence in this region has evolved into a separate branch.

## References

[1] Paton DJ, Brown IH, Edwards S, Wensvoort G (1991). ‘Blue ear’ disease of pigs.. Vet Rec.

[2] Straw BE, Zimmerman JJ, D'Allaire S, Taylor DJ (2006). Diseases of Swine 9th Edition.

[3] Nelsen CJ, Murtaugh MP, Faaberg KS (1999). Porcine reproductive and respiratory syndrome virus comparison: Divergent evolution on two continents.. Journal of Virology.

[4] Jiang P, Chen P, Dong Y, Cai J, Cai B (2000). Isolation and genome characterization of porcine reproductive and respiratory syndrome virus in P.R. China.. J Vet Diagn Invest.

[5] Dea S, Gagnon CA, Mardassi H, Pirzadeh B, Rogan D (2000). Current knowledge on the structural proteins of porcine reproductive and respiratory syndrome (PRRS) virus: Comparison of the North American and European isolates.. Archives of Virology.

[6] Meulenberg JJ, Petersen-den Besten A, De Kluyver EP, Moormann RJ, Schaaper WM (1995). Characterization of proteins encoded by ORFs 2 to 7 of Lelystad virus.. Virology.

[7] Lu J, Gao F, Wei Z, Liu P, Liu C (2011). A 5′ -proximal Stem-loop Structure of 5′ Untranslated Region of Porcine Reproductive and Respiratory Syndrome Virus Genome Is Key for Virus Replication.. Virol J.

[8] Johnson CR, Griggs TF, Gnanandarajah J, Murtaugh MP (2011). Novel structural protein in porcine reproductive and respiratory syndrome virus encoded by an alternative ORF5 present in all arteriviruses.. J Gen Virol.

[9] Mardassi H, Massie B, Dea S (1996). Intracellular synthesis, processing, and transport of proteins encoded by ORFs 5 to 7 of porcine reproductive and respiratory syndrome virus.. Virology.

[10] An T-q, Zhou Y-j, Liu G-q, Tian Z-j, Li J (2007). Genetic diversity and phylogenetic analysis of glycoprotein 5 of PRRSV isolates in mainland China from 1996 to 2006: Coexistence of two NA-subgenotypes with great diversity.. Veterinary Microbiology.

[11] Zhou YJ, Yu H, Tian ZJ, Li GX, Hao XF (2009). Genetic diversity of the ORF5 gene of porcine reproductive and respiratory syndrome virus isolates in China from 2006 to 2008.. Virus Res.

[12] Chen N, Cao Z, Yu X, Deng X, Zhao T (2011). Emergence of novel European genotype porcine reproductive and respiratory syndrome virus in mainland China.. J Gen Virol.

[13] An TQ, Tian ZJ, Leng CL, Peng JM, Tong GZ (2011). Highly pathogenic porcine reproductive and respiratory syndrome virus, Asia.. Emerg Infect Dis.

[14] Mateu E, Díaz I, Darwich L, Casal J, Martín M (2006). Evolution of ORF5 of Spanish porcine reproductive and respiratory syndrome virus strains from 1991 to 2005.. Virus Research.

[15] Tian K, Yu X, Zhao T, Feng Y, Cao Z (2007). Emergence of fatal PRRSV variants: unparalleled outbreaks of atypical PRRS in China and molecular dissection of the unique hallmark.. PLoS One.

[16] Li B, Fang L, Guo X, Gao J, Song T (2011). Epidemiology and Evolutionary Characteristics of the Porcine Reproductive and Respiratory Syndrome Virus in China between 2006 and 2010.. J Clin Microbiol.

[17] Plagemann PGW (2004). The primary GP5 neutralization epitope of North American isolates of porcine reproductive and respiratory syndrome virus.. Veterinary Immunology and Immunopathology.

[18] Ostrowski M, Galeota JA, Jar AM, Platt KB, Osorio FA (2002). Identification of neutralizing and nonneutralizing epitopes in the porcine reproductive and respiratory syndrome virus GP5 ectodomain.. Journal of Virology.

[19] Faaberg KS, Hocker JD, Erdman MM, Harris DLH, Nelson EA (2006). Neutralizing antibody responses of pigs infected with natural GP5 N-glycan mutants of porcine reproductive and respiratory syndrome virus.. Viral Immunology.

[20] Key KF, Guenette DK, Yoon KJ, Halbur PG, Toth TE (2003). Development of a heteroduplex mobility assay to identify field isolates of porcine reproductive and respiratory syndrome virus with nucleotide sequences closely related to those of modified live-attenuated vaccines.. Journal of Clinical Microbiology.

[21] Botner A, Strandbygaard B, Sorensen KJ, Have P, Madsen KG (1997). Appearance of acute PRRS-like symptoms in sow herds after vaccination with a modified live PRRS vaccine.. Vet Rec.

[22] Nielsen HS, Oleksiewicz MB, Forsberg R, Stadejek T, Botner A (2001). Reversion of a live porcine reproductive and respiratory syndrome virus vaccine investigated by parallel mutations.. Journal of General Virology.

[23] Storgaard T, Oleksiewicz M, Botner A (1999). Examination of the selective pressures on a live PRRS vaccine virus.. Arch Virol.

[24] Yang SX, Kwang J, Laegreid W (1998). Comparative sequence analysis of open reading frames 2 to 7 of the modified live vaccine virus and other North American isolates of the porcine reproductive and respiratory syndrome virus.. Archives of Virology.

[25] Zhou L, Chen S, Zhang J, Zeng J, Guo X (2009). Molecular variation analysis of porcine reproductive and respiratory syndrome virus in China.. Virus Res.

[26] Indik S, Valicek L, Klein D, Klanova J (2000). Variations in the major envelope glycoprotein GP5 of Czech strains of porcine reproductive and respiratory syndrome virus.. Journal of General Virology.

